# Morphometric Analysis of the Mental Foramen Using Cone-Beam Computed Tomography

**DOI:** 10.1155/2018/4571895

**Published:** 2018-03-26

**Authors:** Rudyard dos Santos Oliveira, Maria Rodrigues Coutinho, Francine Kühl Panzarella

**Affiliations:** Department of Imaging and Oral Radiology, São Leopoldo Mandic College, Campinas, SP, Brazil

## Abstract

This study evaluated the effects of age and sex on the location and size of the mental foramen (MF). A total of 104 cone-beam computed tomography (CBCT) scans from patients' aged 18–80 years were selected. Images were evaluated using the following parameters: position and size of the MF, and Distances A (distance from the upper limit of the MF to the apex of the first lower premolar), B (distance from the upper cortical border of the MF to the alveolar crest), and C (distance from the border of the MF to the base of the mandible). Results revealed that the location of the MF was predominantly apical (44.4%), between the long axes of the premolars, at an average distance of 4.92 mm from the root of the first lower premolar. The height of the MF was significantly different between both sexes (3.41 and 2.99 mm, resp.; mean height: 3.11 mm; *P*=0.003). The MF was located on average at 11.21 mm from the alveolar crest and 12.31 mm from the base of the mandible; the former measurement was significantly different between both sexes (13.13 and 11.98 mm, resp.; *P* ≤ 0.001). In conclusion, the location of the MF was predominantly apical between the long axes of the premolars, and the mean size and distance of the MF were greater in men.

## 1. Introduction

The mental foramen (MF) is located near the apex of the second premolar teeth, but in an estimated one in four people, the MF is located between the premolars. The MF is usually located midway between the base of the mandible and the alveolar bone crest and in the same vertical plane of the infraorbital foramen. In children, the MF is typically located between the first and second deciduous molars, while in the edentulous elderly, the MF may be located near or on the residual alveolar crest due to the resorption that occurs in the alveolar process in these patients [1].

In recent years, morphometric evaluations of the MF have been of interest; in particular, specialties including endodontics, implantology, and surgery have shown interest in morphometric studies because correct localization of the MF is of paramount importance for procedures such as periapical surgery, incision and flap thickness, and even administration of local anesthetic in this area. In addition, locating the MF is critical for surgical procedures such as removal of blocks for bone grafting, placement of plates for fixation of fractures, mentoplasty, orthognathic surgeries, or during dental implant surgery [2–6].

For example, using periapical radiography, Phillips et al. [7] found that the MF was located approximately 3.8 mm mesial to the apex of the second premolar. The advantage of periapical radiography is that a relatively small amount of radiation is required; however, there can be image enlargement and failure to detect the MF if it is located below the apical edge of the film. Similarly, using computed tomography, Jacobs et al. [8] were able to detect the MF in 100% of cases (*n*=230). Techniques such as computed tomography enable high-resolution imaging and three-dimensional visualization of the surrounding bone and tissues, in addition to being free from excess magnification. However, computed tomography uses larger amounts of radiation compared to periapical and panoramic radiography previously studied.

Nonetheless, Li et al. [9] used computed tomography to retrospectively evaluate 68 Chinese patients in order to identify the anterior inferior alveolar nerve loop and measure its length and position. Results of their study found that the anterior loop could be identified in 83.1% of cases and had an average length of 2.09 mm. The mean distance from the upper border of the MF to the alveolar crest was 13.00 mm, and the mean distance from the upper edge of the origin of the loop anterior to the alveolar crest was 17.83 mm [9].

Similarly, in a retrospective study by Sisman et al. [10], researchers evaluated the location, diameter, area, and the number of accessory MFs. Results found that 14 accessory MFs were observed in 10 (2.0%) of the 504 patients. The frequency found was 2.6% in men and 1.0% in women. The mean distance between the accessory foramen and the MF was 5.0 mm.

In another study using computerized tomography to measure bone thickness around the MF performed by Al-Kalaly et al. [11], the authors reported an average bicortical bone thickness of 10.0–12.0 mm in this region. Ozturk et al. [12] studied the position and location of the MF using cone-beam computed tomography (CBCT). The authors reported that, in most cases, the mandibular canal was located near the lingual cortex or very close (≤2 mm) to it in the molar region. CBCT images from this study showed that the canal moves towards the buccal aspect of the mandible, where it finally emerges through the MF. Three patterns of mandibular canal anatomy were noted: sharp curve (53.2%), smooth curve skirt (28.8%), and straight path (17.4%). In addition, examination of the vertical aspect of the mandibular canal showed that the canal was located almost 1.0 cm above the lower border of the mandible and then rose to reach the MF, which is located 16 mm above the lower border of the mandible.

Recently, von Arx et al. [13] studied the position of the MF and found similar measurements to previous studies using radiography to evaluate the size and position of the MF, as well as the distance between the MF and adjacent anatomical structures. The majority of MFs (56.0%) were located apically between the two premolars, while another 35.7% of MFs were positioned below the second premolar. On average, MFs were located 5.0 mm from the nearest root of the adjacent tooth (range: 0.3–9.8 mm). The mean MF size showed a height of 3.0 mm and a length of 3.2 mm; however, individual cases showed large differences in height (1.8–5.1 mm) and length (1.8–5.5 mm).

Following this reasoning, Carruth et al. [14] conducted a retrospective observational study to determine and compare the size and position of the MF between individuals. The authors reported that 53.7% of MFs were located mesial, 45.3% distal, and 1% coincident with the apex of the second mandibular premolar. Of note, males had significantly higher coronal height and tangential height than females. In addition, black patients had a significantly greater distal horizontal distance from the cementoenamel junction than white patients. The mean width of the MF was 4.08 mm (axial) or 4.12 mm (tangential), while the mean height was 3.54 mm (tangential) or 3.55 mm (coronal).

Among imaging tools that are available for visualizing MF structures, CBCT has proven to be highly valuable, allowing professionals to examine different regions and bone densities of the face in greater detail and fidelity, contributing to more accurate diagnosis, better treatment plans, and more successful treatments, leading to greater patient satisfaction. CBCT also creates three-dimensional images of the maxillofacial mineralized tissues with minimal distortion and significantly reduces radiation exposure for patients [15, 16].

In this context, although the MF is a widely studied anatomical structure, to date, few studies have evaluated the variations of this structure using CBCT. Furthermore, attempts to correlate the location of MF with the patient's sex and age have been rare. Therefore, the objective of this study was to evaluate the effect of age and sex on the location and size of the MF relative to the apices of the adjacent dental elements and the upper and lower borders of the mandible, using CBCT.

## 2. Materials and Methods

This retrospective observational study was approved by our institution's Research Ethics Committee (Protocol no. 52990316500005374). A total of 458 CBCT examinations were available, from which 104 were selected for evaluation after application of the following exclusion criteria: (1) patients with poor bone formation, (2) presence of lesions in the evaluated region, (3) presence of impacted teeth, and (4) lack of premolars in the analyzed region. Of these, 72 (69.2%) were female, and 32 (30.8%) were male. The patients' ages ranged from 18 to 80 years, with a mean age of 49.2 years. Of the 104 CBCT scans, 65 (62.5%) were examined bilaterally, generating a total of 169 measurements (48 MF in male patients and 121 MF in female patients).

All images were acquired with the same protocol using i-CAT tomography (Imaging Sciences International, Hatfield, PA, USA) in 12-bit grayscale and a voxel size of 0.25 mm, allowing for better image definition. Acquired images were analyzed using Xoran software (Xoran Technologies, Ann Arbor, MI, USA) and were reformatted in multiplanar reconstructions to obtain sectors most suitable for the evaluation and measurements of MF.

For standardization of tomographic evaluations, acquired images were analyzed by two previously trained expert radiologists using an intraclass correlation coefficient (ICC) test. Results revealed that intraexaminer reproducibility was excellent both for linear measurements (ICC > 0.9, *P* < 0.0001) and nominal measurements (kappa = 1.0) obtained at two different time points. For both sexes and all age groups, all images were evaluated according to the following parameters: MF position, MF size, Distance A, Distance B, and Distance C.

The sample was divided into four age groups: 18–25 years, 26–40 years, 41–55 years, and ≥56 years. Next, CBCT scans were evaluated to determine the size and position of the MF and to correlate these measurements with the apices of the dental elements. In addition, the distance from the MF to the alveolar crest (Distance B) and the distance from the lower border MF to the lower border of the mandible (Distance C) were measured.

To evaluate the horizontal position of the MF, six locations were analyzed: Position 1 (anterior to the long axis of the first lower premolar (LPM)), Position 2 (in line with the long axis of the first LPM), Position 3 (between the long axes of the first and second LPMs), Position 4 (in line with the long axis of the second LPM), Position 5 (between the long axes of the second LPM and the first molar), and Position 6 (in line with the long axis of the mesial root of the first lower molar).

**Figure 1:**
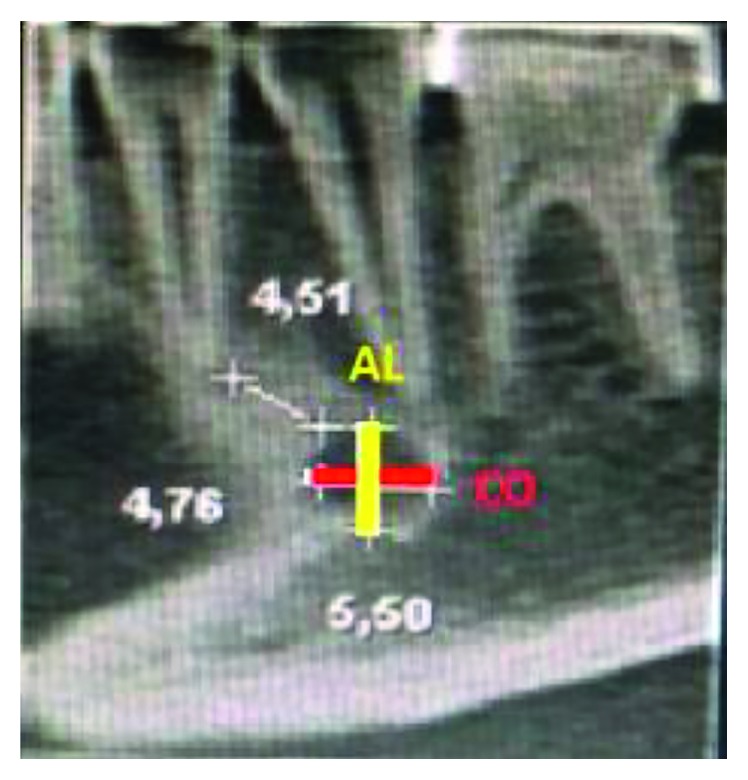
Sagittal view with measurements of the distance to the mental foramen (MF) and MF apex.

A sagittal view selected from the multiplanar reconstructions was also used to better visualize the MF. From this view, a vertical measurement (height, *H*) and a horizontal measurement (length, *L*) were made between the cortical areas of the MF, as shown in Figure 1.

**Figure 2:**
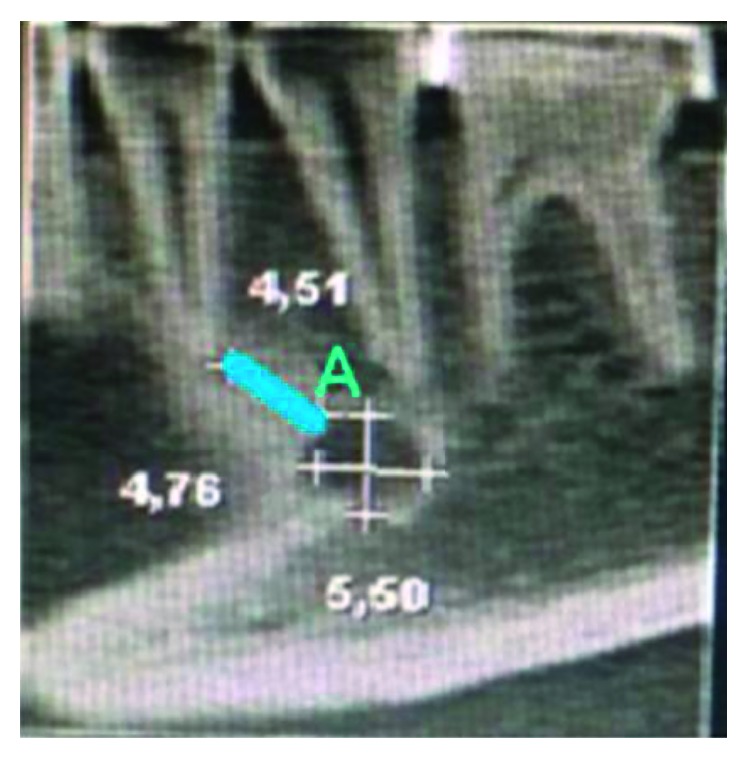
Sagittal view with measurements of the distance from the upper limit of the mental foramen (MF) to the MF apex of the first LPM.

**Figure 3:**
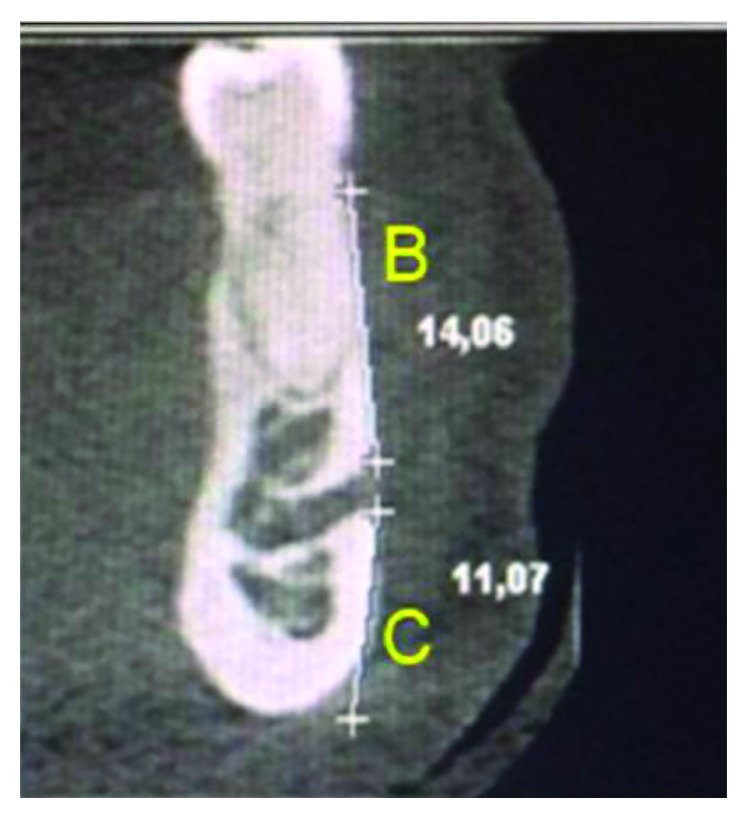
Coronal view with measurements of the mental foramen (MF) outer margin of the alveolar crest and base of the mandible.

The distance from the upper limit of the MF to the apex of the first LPM was designated “Distance A” (Figure 2). The distance from the upper cortical border of the MF to the alveolar crest was designated “Distance B.” This distance was measured by selecting the coronal slice that best identified the MF opening in the vestibular margin (Figure 3). The distance from the border of the MF to the base of the mandible was designated “Distance C.” This distance was analyzed in the coronal view to identify the area corresponding to the opening of the MF, and from there, the distance from the external border of the MF to the alveolar crest (Distance B) to the base of the mandible (Distance C) was measured (Figure 3).

Possible differences in these measurements between the age groups and between the sexes were evaluated by adjusting mixed linear models and applying the likelihood ratio test. The use of mixed linear models was necessary because of the presence of repeated measurements. These repeated measures occurred because both sides of the face were examined in some patients, generating two measures (e.g., two height measures) for a given patient. Measurements performed on separate sides of the face but in the same patient are not independent, and this dependence is needed to be considered in the analysis. Therefore, we opted to use mixed linear models, which take into account the presence of repeated measurements in the data, rather than traditional methods (such as variance analysis and the Kruskal–Wallis test), which assume that all observations in the dataset are independent.

Fisher's exact test was used to evaluate the association between MF position, age, and sex. Results are presented below. The level of significance was 5% for all applied tests. All analyses were conducted using R software, version 3.2.2 (www.r-project.org).

## 3. Results

The frequency distribution of measurements in the total sample according to sex and age groups is shown in Table 1.

The distribution of MF locations according to sex is shown in Table 2. There were no significant differences between the sexes (*P* < 0.3246).

The distribution of MF locations according to age groups is shown in Table 3. There were no significant difference between the age groups (*P* < 0.6236).

With regard to the location of the MF, 75 MFs (44.4%) were located between the long axes of the first LPM and second LPM (Position 3), 69 MFs (40.8%) were located below the second LPM (Position 4), 18 MFs (10.7%) were located in line with the long axis of the first LPM (Position 2), and 5 MFs (3.0%) were found between the long axes of the second LPM and the first lower molar (Position 5). Only one MF (0.6%) was located anterior to the long axis of the first LPM and in line with the long axis of the mesial root of the first lower molar (Positions 1 and 6, resp.). There was no significant difference in MF location between the sexes (*P*=0.3246) and age groups (*P*=0.6236).

The average MF height was 3.11 mm (range: 1.27–5.55 mm) (Table 4). There was a significant difference in the average MF height between the sexes (*P*=0.0037), and the average values were higher in male patients than in female patients (3.41 mm in men and 2.99 mm in women). However, there was no significant difference in this parameter between the age groups (*P*=0.1094). No significant difference in the MF length was found between the sexes (*P*=0.2682) or between the age groups (*P*=0.3058) (Table 5).

Distance A was defined as the distance from the uppermost limit of the MF to the apex of the first LPM. The average value was 4.92 mm (range: 1.00–12.75 mm) in both sexes.  Table 6 presents the values for Distance A. No significant differences were found at *α* = 5% level between the sexes (*P*=0.6861) or between the age groups (*P*=0.4709).

In the coronal view, the average distance from the upper border of the MF to the alveolar crest (Distance B) was 11.21 mm (range: 5.01–17.52 mm). No significant differences in this measurement were found at the 5% level between the sexes (*P*=0.9003) or between the age groups (*P*=0.5899) (Table 7).

The lower border of the MF was located 12.31 mm above the lower border of the mandible (Distance C). The values for Distance C are shown in Table 8. A significant difference in this distance was found between the sexes (*P* ≤ 0.001) (13.13 mm in men and 11.98 mm in women) but not between the age groups (*P*=0.6814).

## 4. Discussion

Studies show that correctly locating the MF is important for the success of many dental procedures involving the mandible, including the placement of anesthesia during routine dental, endodontic, periodontic, and dental procedures for children and more invasive procedures such as orthognathic surgeries and implant placement, among others [4, 13, 17–19].

Dental surgeons should be aware that the MF can vary in shape, size, and number and has different vertical and horizontal positions. In addition, the MF can be confused with pathological lesions. For these reasons, several authors have emphasized the importance of studying the location of the MF, thereby avoiding severe lesions to the neurovascular bundle present at this site [13, 16, 19–21].

In the present study, the position of the MF was predominantly apical and was located either between the long axes of the first and second LPMs (44.4%) or below the second LPM (40.8%). These results agree with results from other recent studies (56.0% and 35.7%, resp.) [13]. Similar results were obtained using CBCT (59.8% and 30.4%, resp.) [15, 22].

The average height and length of the MF in this study (3.11 mm and 3.20 mm) were also similar to values reported by von Arx et al. [13] using CBCT (3.0 mm and 3.2 mm, resp.) and values reported by Kalender et al. [15] (3.7 mm and 3.4 mm, resp.). In addition, both von Arx et al. [13] and Kalender et al. [15] found that the MF was larger in men than in women. This difference between the sexes was also found in the present study for the MF height in sagittal CBCT images, and men presented higher average values than women (3.41 and 2.99 mm, resp.).

In this study, we found no significant differences in the length of MF between the sexes. There was also no significant difference in the size of MF between the age groups in this study, as in some recent studies [13, 22].

In this study, we defined Distance A as the distance from the uppermost limit of the MF to the apex of the first LPM. The average value of Distance A for both sexes was 4.9 mm distally from the first LPM. We found no significant difference in this measurement between the sexes or between the age groups. We used the apex of the first LPM as a parameter for this measurement because this is a significant landmark for dental anesthesia, suggesting that the dentist should position the anesthetic needle between the two LPMs but at an average distance of 4.9 mm distal to the apex of the first LPM. Moreover, this parameter was useful from the surgical point of view, as a means to avoid damage to the neurovascular bundle. Our finding agrees with results of von Arx et al. [13], which found an average distance from the MF to the nearest root of 5 mm, and with findings of Kalender et al. [15], which found an average distance from the MF to the nearest tooth of 4.0 mm.

In the coronal view, the average distance between the upper border of the MF and the alveolar crest (Distance B) was 11.2 mm (range: 5.0–17.5 mm). We did not find statistically significant differences in this parameter between the sexes or between the age groups. In the samples used in recent studies [2, 5, 9], the average distance from the MF to the alveolar crest was 12.6 mm, and the range was 5.7 to 19.6 mm. The values found in these studies were similar to those observed in this study, but the variability in measurements suggests that this distance cannot be consistent because of the loss of alveolar bone.

For Distance C, the lower border of the MF was located 12.3 mm (range: 8.5–16.2 mm) above the lower border of the mandible. We found a significant difference between the sexes (13.1 mm in men and 11.9 mm in women), but not between the age groups. Previous studies have reported that the MF was located 13.2 mm above the lower border of the mandible, with significant sex differences (men tended to have higher values than women) [13, 15, 22–24].

The average distance found by Kalender et al. [15] was 12.4 mm. Kalender et al. also found significant differences between the sexes: values were higher in men (average of 13.1 mm) than in women (average of 11.8 mm). These results and the discovery that the average distance from the MF to the apex of the first LPM was 4.92 mm should be of significant benefit for surgeons needing to anesthetize the mental nerve in surgeries in this region. In addition, we believe that the calculations of the average MF size, average distance between the MF and the alveolar crest, and average distance between the MF and the base of the mandible produced in this study will aid dental surgeons in clinical analysis and be useful as a parameter for future interventions [25, 26].

## 5. Conclusions

In the present study, we determined that the position of the MF was predominantly apical and was located between the long axes of the first lower premolar and the second lower premolar and, on average, 4.9 mm from the apex of the first premolar bottom. In addition, the average MF size and the average distance between the MF and the base of the mandible were higher in men, and there was no significant difference between the age groups.

## Figures and Tables

**Table 1:** Sample frequency distribution by sex and age.

Age group (years)	Sex		Total *N* (%)
	Female	Male	
	*n* (%)	*n* (%)	
18–25	12 (9.9)	4 (8.3)	16 (9.5)
26–40	24 (19.8)	9 (18.8)	33 (19.5)
42–55	54 (44.6)	16 (33.3)	70 (41.4)
≥56	31 (25.6)	19 (39.6)	50 (29.6)

Total	121 (100.0)	48 (100.0)	169 (100.0)

**Table 2:** Distribution of mental foramen positions for the sample by sex.

Position	Sex		Total *N* (%)
	Female	Male	
	*n* (%)	*n* (%)	
1	1 (0.8)	0 (0.0)	1 (0.6)
2	15 (12.4)	3 (6.3)	18 (10.7)
3	57 (47.1)	18 (37.5)	75 (44.4)
4	43 (35.5)	26 (54.2)	69 (40.8)
5	4 (3.3)	1 (2.1)	5 (3.0)
6	1 (0.8)	0 (0.0)	1 (0.6)

Total	121 (100.0)	48 (100.0)	169 (100.0)

**Table 3:** Distribution of mandibular foramen positions for the sample by age.

Position	Age group (years)				
	18–25	26–40	42–55	≥56	Total
	*n* (%)	*n* (%)	*n* (%)	*n* (%)	*N* (%)
1	0 (0.0)	0 (0.0)	1 (1.4)	0 (0.0)	1 (0.6)
2	2 (12.5)	4 (12.1)	9 (12.9)	3 (6.0)	18 (10.7)
3	7 (43.8)	17 (51.5)	24 (34.3)	27 (54.0)	75 (44.4)
4	7 (43.8)	12 (36.4)	32 (45.7)	18 (36.0)	69 (40.8)
5	0 (0.0)	0 (0.0)	4 (5.7)	1 (2.0)	5 (3.0)
6	0 (0.0)	0 (0.0)	0 (0.0)	1 (2.0)	1 (0.6)

Total	16 (100.0)	33 (100.0)	70 (100.0)	50 (100.0)	169 (100.0)

**Table 4:** Analysis of height measurements.

Height								
Group	Minimum	1st quartile	Median	Average	3rd quartile	Maximum	Standard deviation	*P* value^∗^
All	1.27	2.55	3.00	3.11	3.54	5.55	0.75	

Female	1.75	2.51	2.85	2.99	3.26	5.01	0.63	0.0037
Male	1.27	2.76	3.25	3.41	3.96	5.55	0.93	

18–25	2.30	2.54	3.40	3.45	4.01	5.55	1.04	0.1094
26–40	1.75	2.75	3.25	3.27	3.95	5.01	0.85	
42–55	1.27	2.61	2.78	2.99	3.33	5.51	0.64	
≥56	1.86	2.50	3.00	3.07	3.45	4.75	0.60	

^∗^ *P* value was obtained from the likelihood ratio test.

**Table 5:** Analysis of length measurements.

Length								
Group	Minimum	1st quartile	Median	Average	3rd quartile	Maximum	Standard deviation	*P* value^∗^
All	1.50	2.51	3.15	3.20	3.76	5.55	0.88	

Female	1.75	2.50	3.04	3.14	3.70	5.50	0.89	0.2682
Male	1.50	2.64	3.25	3.32	4.00	5.55	0.86	

18–25	2.00	2.30	3.15	3.19	3.50	5.55	1.05	0.3058
26–40	1.75	3.00	3.26	3.46	4.00	5.50	1.01	
42–55	1.50	2.51	3.13	3.17	3.76	5.50	0.84	
≥56	2.00	2.50	3.01	3.07	3.55	5.01	0.78	

^∗^ *P* value was obtained from the likelihood ratio test.

**Table 6:** Analysis of measurements of Distance A.

Distance (mm)								
Group	Minimum	1st quartile	Median	Average	3rd quartile	Maximum	Standard deviation	*P* value^∗^
All	1.00	3.26	4.74	4.92	6.40	12.75	2.22	

Female	1.00	3.26	4.78	4.92	6.34	12.75	2.15	0.6861
Male	1.23	3.21	4.55	4.92	6.76	11.16	2.42	

18–25	1.42	3.32	4.74	4.55	5.71	8.33	1.96	0.4709
26–40	2.76	3.76	5.48	5.36	6.45	8.88	1.75	
42–55	1.00	3.20	4.51	4.90	6.59	10.64	2.20	
≥56	1.55	2.75	4.16	4.76	6.07	12.75	2.60	

^∗^ *P* value was obtained from the likelihood ratio test.

**Table 7:** Analysis of measurements of Distance B.

Distance (mm)								
Group	Minimum	1st quartile	Median	Average	3rd quartile	Maximum	Standard deviation	*P* value^∗^
All	5.01	9.95	11.35	11.21	12.50	17.52	1.99	

Female	5.01	9.78	11.50	11.18	12.52	15.55	1.99	0.9003
Male	7.25	10.06	11.27	11.29	12.26	17.52	1.99	

18–25	8.98	10.89	12.18	11.99	13.07	14.58	1.59	0.5899
26–40	6.54	9.28	11.25	11.01	12.26	14.63	1.89	
42–55	5.76	9.76	11.29	11.17	12.60	15.55	2.01	
≥56	5.01	10.02	11.36	11.15	12.20	17.52	2.13	

^∗^ *P* value was obtained from the likelihood ratio test.

**Table 8:** Analysis of measurements of Distance C.

Distance (mm)								
Group	Minimum	1st quartile	Median	Average	3rd quartile	Maximum	Standard deviation	*P* value^∗^
All	8.50	11.07	12.31	12.31	13.51	16.26	1.66	

Female	8.50	10.98	12.02	11.98	13.10	16.26	1.58	0.0001
Male	10.08	11.95	13.26	13.13	14.28	16.25	1.57	

18–25	9.73	10.87	11.50	11.68	12.62	14.74	1.31	0.6814
26–40	9.55	11.16	12.50	12.32	13.19	15.55	1.46	
42–55	8.50	11.25	12.36	12.32	13.52	16.26	1.76	
≥56	9.51	11.02	12.60	12.48	13.75	16.25	1.72	

^∗^ *P* value was obtained the from likelihood ratio test.
